# How to Clean Old Slides and Utilize Spoiled Mounts

**Published:** 1891-09

**Authors:** H. M. Whelpley


### How to Clean Old Slides and Utilize Spoiled Mounts.

Dr. H. M. Whelpley, F.R.M.S., writes: For two years past I have permitted soiled slides and spoiled mounts to accumulate in a box set aside for that purpose. The process I have recently followed in reclaiming them has been successful. I first placed the unsightly rubbish in a dish of clean water, where it remained until all the labels were readily removed. With an old knife I next scraped off the cells and all cement that could be easily removed in this manner. All slides where glycerine or other substance soluble in water had been used as a mounting medium were again washed, and then the entire pile spread out and dried. I separated those that were clean and placed the rest in alcohol for several days. This solvent cleaned another portion of the slides so that all they required to render them as good as new was a washing in water. The remaining dirty ones were treated to a bath of oil of turpentine, where they rested for a few days. From this they were washed with alcohol and then finshed in water. The few refractory ones that held out during all this time were made as clean as ever with benzol.

Although considerable time elapsed before the last slide was cleaned, it required but a few minutes of actual labor in the entire process. The time consumed is in letting them stand in the different liquids. Nor is the process expensive, as the oil of turpentine did most of the work. Hereafter I shall divide my old slides into three classes and clean them separately, so that less alcohol will be required. The first box will contain slides that can be washed clean with water, the second lot will be those that alcohol will clean, and the third one requiring benzol. Cover-glasses are so cheap that I do not save them unless they are easily cleaned with water. I find it very difficult to properly clean thin cover-glasses that have cement on them.—*Amirican Microscopical Journal.*

---

TOOTHACHE DROPS.—A novel toothache tincture is recommended. It is composed of 2 drops of pure coniine and 8 drops of oil of cinnamon dissolved in 4 drops of rectified spirit. A single drop of the tincture on cotton wool placed in the tooth is said to give splendid results.—*Druggists' Bulletin.*

